# Autoantibodies reactive with glomerular endothelial cells and podocytes in patients with membranous nephropathy

**DOI:** 10.1016/j.jtauto.2025.100342

**Published:** 2025-12-09

**Authors:** Vojtech Petr, Shrey Purohit, Felix Poppelaars, Brandon Renner, Jennifer Laskowski, Russell Whelan, Liudmila Kulik, Jessica Kendrick, Ashley Frazer-Abel, Diana Jalal, Barbara Marcolin, Isabelle Schmelzer, Hanna Debiec, Pierre Ronco, Moin A. Saleem, Simon C. Satchell, Joshua M. Thurman

**Affiliations:** a Institute for Clinical and Experimental Medicine, Prague, Czech Republic; b Department of Medicine, Anschutz Medical Campus, University of Colorado School of Medicine, Aurora, CO, USA; c Exsera BioLabs, Anschutz Medical Campus, University of Colorado School of Medicine, Aurora, CO, USA; d Department of Medicine, Carver College of Medicine, University of Iowa, IA, USA; e Iowa City VA HCS, Iowa City, IA, USA; f Department of Nephrology and Dialysis, Sorbonne University, Paris, France; g Department of Pediatric Nephrology, Bristol Renal and Royal Bristol Children Hospital, University of Bristol, Bristol, UK; h Bristol Renal, University of Bristol, Bristol, UK

**Keywords:** Membranous nephropathy, Complement, Autoantibody, Glomerular endothelial cells

## Abstract

**Rationale & objective:**

Membranous nephropathy (MN) is a glomerular disease caused by autoantibodies reactive with podocyte antigens. The most common antigen is the M-type phospholipase A2 receptor (PLA2R), but autoantibodies to other podocyte antigens have also been identified. Investigators have reported elevated levels of complement fragments in plasma. However, most complement fragments generated on podocytes are likely to pass into the urine and not enter the bloodstream. Further, anti-PLA2R antibodies are usually IgG4 subclass and do not activate the classical pathway of complement. To look for additional autoantibodies capable of generating endovascular complement fragments, we examined whether MN patients have antibodies reactive with endothelial cell antigens.

**Study design:**

Retrospective cohort study.

**Setting & participants:**

We analyzed plasma samples from 64 patients with MN, and results were compared to healthy controls and patients with chronic kidney disease.

**Exposure:**

Plasma and urine complement activation fragments, glomerular endothelial cell and podocyte antibody binding assays, anti-cardiolipin antibody enzyme linked immunosorbent assay.

**Outcome:**

Proteinuria, estimated glomerular filtration rate.

**Analytical approach:**

Groups were compared with Wilcoxon, Kruskal-Wallis or chi-square tests. Correlations were performed using Pearson's correlation.

**Results:**

Plasma C3a, C4a, C5a, and sC5b-9 levels were elevated in MN patients. Some patients had IgG reacted with glomerular endothelial cells or with podocytes. These antibodies were seen in distinct subsets of patients and did not correlate with the presence of anti-PLA2R antibodies. Higher titers of anti-glomerular endothelial cell antibodies correlated with systemic complement activation, seen by sC5b-9, and disease severity, determined by proteinuria. Anti-cardiolipin IgG levels associated with proteinuria.

**Limitations:**

Assays used immortalized cell lines, and target antigens have not yet been identified.

**Conclusions:**

MN is a disease of autoimmunity directed against podocyte antigens, but some patients may also produce autoantibodies that target antigens on glomerular endothelial cells. The level of these antibodies correlates with adverse clinical findings.

## 1 Introduction

Primary membranous nephropathy (MN), an autoantibody-mediated glomerular disease, is the most common cause of nephrotic syndrome in adults [1]. Patients generally present with edema, hypoalbuminemia, and mild kidney dysfunction, but the natural course of the disease is highly variable. Approximately one third of patients have spontaneous remission with supportive care, whereas another one third of patients will have progressive disease if untreated [2]. Given the heterogeneity in patient outcomes, prognostic biomarkers are important for identifying which patients are most likely to benefit from immunosuppressive treatment. In addition, the clinical response to treatment with immunosuppressive medications is 50–60 %, so there is also still a need to identify new therapeutic targets [3,4].

Immunofluorescence microscopy of biopsy tissue from patients with primary MN typically reveals deposits of granular IgG in the glomerular capillaries, and electron microscopy shows that the deposits are primarily restricted to the subepithelial region [5]. The most common target antigen in adult primary MN is the M-Type phospholipase A2 receptor (PLA2R), and antibodies reactive with this podocyte protein are present in 80 % of cases of MN [6]. These antibodies are usually IgG4, an isotype that does not activate the classical pathway of complement, although it may activate the lectin pathway [7,8]. Glomerular C3 deposits are seen in most cases, however, indicating that complement activation is an intrinsic part of the disease process [9]. Because the immune deposits are adjacent to podocytes within glomerular capillaries, most of the complement activation fragments generated are probably lost in the urine, explaining why the kidneys of MN patients are not infiltrated by inflammatory cells [10]. However, detection of complement fragments in urine, particularly C5b-9, may be a useful way of monitoring immunologic activity [11]. Furthermore, one study reported that elevated levels of complement fragments can be detected in plasma of some patients [12]. This finding suggests that complement fragments generated in the subepithelial space can migrate back into the bloodstream against the direction of filtration, or that glomerular inflammation in MN also involves ultrastructural locations with access to the circulation.

While studying focal segmental glomerulosclerosis (FSGS), we discovered that natural IgM binds to neoepitopes displayed on the surface of injured glomerular endothelial cells GEnCs; [13]. We also found that plasma complement proteins were elevated in FSGS patients, consistent with endovascular complement activation by the antibodies. Natural antibodies are present in all individuals from birth, although the repertoires vary among individuals [13,14]. We hypothesized, therefore, that natural antibodies may be a common downstream cause of secondary disease progression after many types of glomerular injury, and that variation in the titers of natural antibodies may contribute to the heterogeneity of the prognosis in MN. Furthermore, autoantibodies reactive with endothelial antigens could increase the concentration of complement fragments in plasma. In the current study we first confirmed that complement fragments are elevated in plasma of MN patients. We then investigated whether antibodies reactive with GEnCs, podocytes or cardiolipin were present in MN samples, and whether they correlated with the levels of complement activation fragments and clinical outcomes.

## 2 Methods and materials

### 2.1 Plasma, serum, tissue and urine samples

We analyzed plasma, serum, and urine samples from 64 MN patients, 42 of whom had anti-PLA2R antibodies (Table 1). As controls, we analyzed samples from 30 healthy patients and 30 patients with chronic kidney disease (Supplementary Table 1). Clinical characteristics of the CKD patients have been previously reported [15]. We also analyzed human glomerular extracts that were generated as previously described (generously provided by David J. Salant) [6]. All samples were obtained with approval of the Institutional Review Board (protocol number: 14–1209), the Human Subjects Committee and all subjects consented to use of these samples for research purposes.

**Table 1:** Participant demographics.

Characteristic	N = 64
Male sex, n (%)	45 (71 %)
Age, median (IQR), [years]	58 (48, 68)
Autoantibodies	•42 Anti-PLA2R (69 % of tested) •1 Anti-THSD7A (2 % of tested) •18 Negative (30 % of tested) •3 Not performed
Serum creatinine, median (IQR), [mg/dl]	1.18 (0.84, 2.17)
Estimated glomerular filtration rate, median (IQR), ml/min/1.73m^2^	60 (30, 84)
Urinary protein-creatinine ratio, median (IQR), [mg/g]	560 (340, 975)
Urinary albumin-creatinine ratio, median (IQR), [mg/g]	457 (252, 681)
Primary membranous nephropathy, n (%)	45 (83 %)

### 2.2 Complement ELISAs

Complement fragments were detected in patient plasma and urine using MicroVue enzyme immunoassay kits (Quidel). Plasma was diluted 1:200 (C3a), 1:40 (C4a), 1:20 (C5a), 1:10 (sC5b-9), and 1:1000 (Ba). Urine was diluted 1:5 or 1:10 (for healthy controls and CKD group or MN group, respectively) for C4a and sC5b-9 analysis, and 1:200 (Ba). Assays were performed, and analyte concentrations were determined according to manufacturer specifications. Complement fragments C3a, C4a, C5a, factor H, and sC5b-9 in the MN plasma samples were detected using the Q-Plex multiplex ELISA (Quansys Biosciences). Plasma was diluted 1:100 and assayed according to manufacturer instructions. Upon completion, the plates were imaged using the chemiluminescent Q-View Imager Pro (Quansys Biosciences). Data were analyzed and final analyte concentrations were calculated using the Q-View software package (Quansys Biosciences). Urine complement fragments were not analyzed using the Q-Plex system because the various analytes required different dilutions and could not be run as a panel.

### 2.3 Cell binding assays

We used an *in vitro* cell-binding assay to measure binding of IgG or IgM in serum to an immortalized podocyte cell line that has previously been reported to not express PLA2R [16,17] and an immortalized glomerular endothelial cell line [18]. Cells were cultured on 24-well plates until they reached 80–90 % confluence. Media was removed and the cells were washed with 1 % bovine serum albumin (BSA) in PBS. Media containing 10 % patient serum was then added to each well, and the cells were incubated at 37 °C for 1 h. Cells were then washed, detached from the plate with 100 μl of Accutase (Innovative Cell Technologies) and resuspended in 1 % BSA in PBS. To detect IgG and IgM bound to the cells, they were resuspended in 100 μl of mouse anti-human IgG Fc-Alexa Fluor 647 (Southern Biotech) or goat anti-human IgM, μ-chain (Abcam). The cells were incubated at 4 °C for 1 h while covered from light and then washed. To test for the presence of PLA2R on the surface of differentiated podocytes mouse anti-human PLA2R (Sigma Aldrich) was used in place of serum in the above-described protocol. The secondary antibody was a goat anti-mouse conjugated to FITC (Abcam). After the cells were washed, they were resuspended in 200ul of 1 % BSA in PBS in round bottom flow cytometry tubes. Flow cytometry was preformed using Cytek Aurora (Cytek® Biosciences). Data was analyzed using FlowJo™ v10.10 Software (BD Life Sciences).

### 2.4 Immunofluorescence staining for IgG binding on podocytes and GEnCs

To detect IgG binding by microscopy, differentiated podocytes and GEnCs were cultured to 80–90 % confluence on 35 mm glass bottom plates (MatTek). The cells were then rinsed twice with warm PBS and fixed with 4 % paraformaldehyde at 25 °C for 10 min, and then they were rinsed with 4 °C PBS and washed twice for 5 min with cold PBS. Non-specific binding of antibodies to the cells was blocked with 5 % fetal bovine serum and 2 % BSA in PBS for 1 h at room temperature. The cells were then rinsed with cold PBS and incubated with 10 % serum from MN patients or control groups overnight at 4 °C. Cells were washed three times with cold PBS, and antibody bound to the cells was detected with mouse anti-human IgG Fc-Alexa Fluor 647 (Southern Biotech) diluted in 2 % FBS and 1 % BSA. Cells were incubated for 1 h while protected from light and then rinsed with cold PBS. 4′,6-diamidino-2-phenylindole, dihydrochloride (1:5000 DAPI; Invitrogen; Thermo Fisher Scientific, Inc) was added in PBS as a nuclear counterstain and cells were incubated for 5 min in the dark. Cells were then washed twice with cold PBS for 5 min. A 50 % glycerin/PBS solution was used to mount the slides. Imaging was performed with a Keyence BZ-X800E microscope (Keyence Corp.).

### 2.5 Anti-cardiolipin antibodies ELISA

Levels of anti-cardiolipin (aCL) IgG and IgM were tested with as described previously [19]. In brief, plates were coated with 50 μg/mL cardiolipin from bovine heart (Avanti) for IgM, and 25 μg/mL for IgG, diluted in high performance liquid chromatography (HPLC) grade ethanol. Plates were blocked with 10 % non-heat inactivated fetal bovine serum (FBS; HyClone) in PBS for 1 h at room temperature. Standards and plasma samples were diluted in blocking buffer and incubated for 1 h at 4 °C. Human aCL IgG and IgM standards were purchased from Louisville APL Diagnostics (Louisville, GA) and prepared according to the manufacturer's instructions. Horseradish peroxidase-conjugated goat anti-human antibodies (EMD Millipore (cat # AP114P) and Invitrogen (cat # A18805)) were then used to detect IgM and IgG. Each step was followed by washing with PBS. Then, 3,3′,5,5′-tetramethylbenzidine was added and the reaction was terminated with 0.2 M sulfuric acid. The optical density was measured at 450 nm. A log-log calibration curve was generated using background adjusted mean values of human standards and was used to determine the levels of aCL antibodies (μg/mL). Non-heat inactivated FBS is used to provide co-factor for aCL antibody binding.

### 2.6 Western blot analysis

Whole glomerular extracts were generated as previously described [6]. Endothelial cells were lysed on ice for 20 min in a buffer containing 0.5 % Triton X-100, 0.5 % Chaps, 20 mM Tris-HCl (pH 7.5), 0.5 M NaCl, 1 mM EDTA, 10 μg/ml leupeptin, and a protease and phosphatase inhibitor cocktail (Sigma). Lysates were cleared by centrifugation at 8000×*g* for 5 min. Lysates were diluted with 4X NuPage LDS sample buffer unreduced samples. For reduced samples, 2-mercaptoethanol was added to the samples and they were boiled for 5 min. The samples were then separated on a NuPAGE 4–12 % Bis-Tris gel. The proteins were transferred to polyvinylidene difluoride (PVDF) or nitrocellulose membranes using Tris-Tris/glycine, pH9.4–10.4. The membranes were blocked overnight with 5 % non-fat milk dissolved in PBS. The membrane was washed in PBS and then probed with pooled serum from five MN patients who had shown anti-GEnC reactivity. The serum was diluted 1:1000 in Western blot buffer (2 % milk/PBS/0.1 %/tween-20). The blots were then washed with buffer and incubated with horseradish peroxidase-conjugated secondary antibodies.

The blots were also re-probed with an antibody against Beta actin (SIgma), or anti-human PLA2R (Sigma). Positive signals were visualized using the ECL system (PerkinElmer).

### 2.7 Statistical analysis

Statistical analysis was performed using R version 4.4.0 and GraphPad Prism version 10.3.1 (Boston, MA). Continuous variables are reported as medians and interquartile ranges (IQR), categorial values as proportions. Groups were compared with Wilcoxon, Kruskal-Wallis or chi-square tests as appropriate. Post hoc analysis for the Kruskal-Wallis test were performed with Dunn's test. Correlations were evaluated using Pearson's correlation. Positive binding to GEnCs or podocytes was defined as mean fluorescence intensity (MFI) above average + 2 standard deviations of MFI from healthy controls.

## 3 Results

### 3.1 Higher systemic complement activation is seen in patients with MN compared to controls

**Fig. 1:**
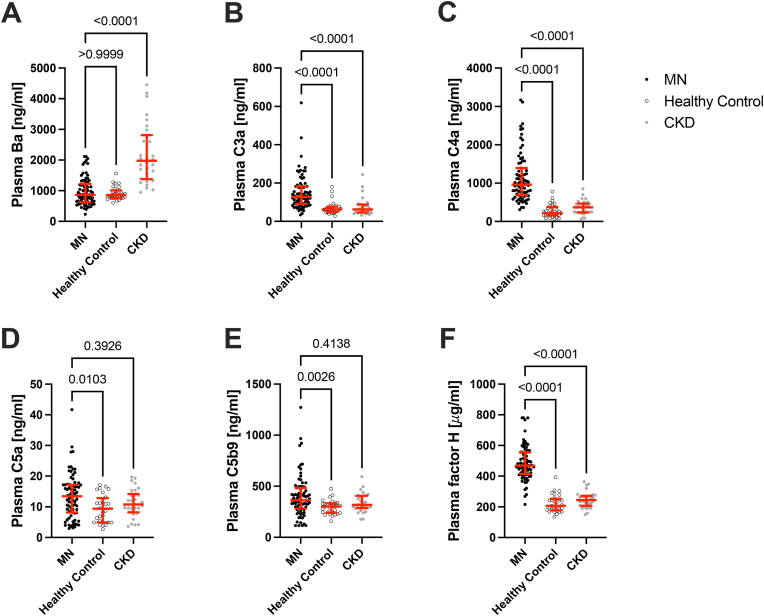
**Complement fragments in plasma from patients with membranous nephropathy.** We measured complement activation fragments in EDTA plasma samples from patients with active membranous nephropathy, including A) Ba, B) C3a, C) C4a, D) C5a, and E) soluble C5b-9. We also measured factor H (F), a soluble complement regulator. The levels of C3a, C4a, and C5a were significantly higher in the plasma of patients with membranous nephropathy than in healthy controls or patients with chronic kidney disease (CKD). Ba levels were similar in the membranous nephropathy samples and in healthy controls, but they were higher in patients with CKD. Factor H levels were higher in the membranous nephropathy samples than in either of the control groups. The horizontal red lines show the median values for each group, and whiskers denote the interquartile range.

We analyzed complement activation fragments in EDTA plasma samples from patients with MN and compared them to the levels in healthy and CKD controls (Fig. 1). We measured C4a as a marker of classical and lectin pathway activation, Ba as a marker of alternative pathway activation, and sC5b-9 as a marker of terminal complement activation, we also measured C3a, C5a, and factor H. Plasma levels of Ba (Fig. 1A) were highest in the CKD samples, consistent with previous reports showing that a decline in kidney function is associated with alternative pathway activation [15,20]. Ba levels were significantly higher in CKD compared to MN regardless of similar kidney function and higher proteinuria in MN (Supplementary Fig. 1). Plasma levels of C3a and C4a were higher in MN than in CKD and healthy controls (Fig. 1B–C). C5a and C5b-9 levels (Fig. 1D–E) were higher in MN than in healthy controls, but similar to the CKD group. Taken together, these findings indicate systemic complement activation in MN patients with predominance of classical/lectin and terminal pathway. Surprisingly, factor H was significantly higher in MN patients than in either healthy or CKD subjects (Fig. 1F), which may explain lower Ba in MN than in CKD subjects. The presence or absence of detectable anti-PLA2R antibodies did not correlate with the level of any of the complement activation fragments (Supplementary Fig. 2 A-F).

We also measured C4a, Ba, and sC5b-9 in the urine of the MN samples, and compared the results to levels in healthy and CKD controls (Supplementary Fig. 3). Urinary sC5b-9 levels were significantly higher in the urine from MN patients compared to healthy and CKD controls, similar to what has been reported previously [21,22]. C4a and Ba levels in the urine were not significantly different in MN compared to healthy individuals.

### 3.2 A subset of MN patients has IgG antibodies that bind to glomerular endothelial cells *in vitro*

**Fig. 2:**
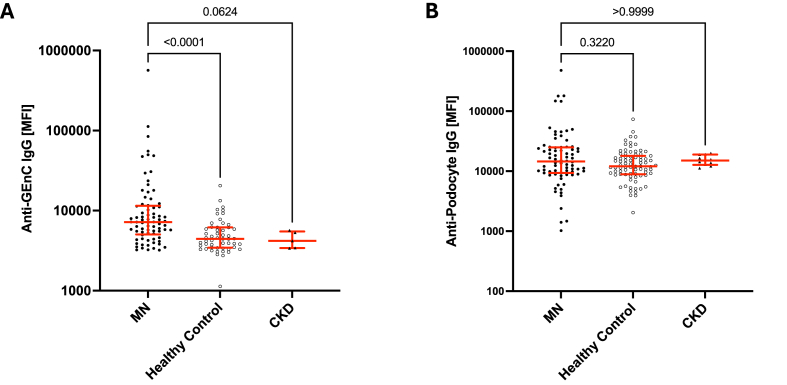
**Anti-glomerular endothelial cell and anti-podocyte antibodies in plasma from patients with membranous nephropathy.** We examined whether IgG and IgM in plasma from patients with membranous nephropathy (MN) would bind to A) glomerular endothelial cells (GEnCs) or B) podocytes *in vitro*. Levels of IgG that bound to GEnCs were significantly higher in the MN samples than in healthy and chronic kidney disease (CKD) controls. Binding of IgG to the podocytes was similar in all of the groups. The horizontal red lines show the median values for each group, and whiskers denote the interquartile range.

We have previously found that natural antibody IgM binds to epitopes displayed on injured endothelial cells [13]. To test whether anti-endothelial antibodies are present in MN patients, we incubated immortalized human GEnC cells with serum from MN patients, healthy controls, and patients with CKD (Fig. 2A). Approximately 20 % of MN samples showed IgG binding to the GEnCs by flow cytometry, and the MFIs for the MN samples (median 7198, IQR 5046, 11504) were significantly higher than those in healthy controls (median 4431, IQR 3446, 6172) and CKD controls (median 3793, IQR 3522, 5311). IgM binding to GEnCs was tested in a subset of patients with MN (n = 7) and healthy controls (n = 13), no difference was found (median 6682, IQR 6362, 9631 vs. median 6528, IQR 5953, 8016; p = 0.438). There was no association between the presence of anti-PLA2R antibodies and anti-GEnC reactivity in these assays (Supplementary Fig. 2G).

### 3.3 A separate subset of MN patients have IgG antibodies that bind to podocytes *in vitro*

We next incubated immortalized human podocytes, that do not express PLA2R, with MN and control serum samples, and examined binding of IgM and IgG to the cells by flow cytometry (Fig. 2B). We first confirmed that these podocytes do not express PLA2R by immunostaining them with a commercial anti-PLA2R antibody at varying dilutions, (Supplementary Fig. 4). Using serum samples of the different groups, we found that 13 % of the MN samples showed IgG binding to podocytes, however, the median MFI for IgG-binding to podocytes from MN samples was not significantly different from those of control samples (14515, IQR 9367, 24947 vs 12056, IQR 8897, 18064). This suggests that the anti-podocyte IgG, when present, binds to antigens other than PLAR2 on this cell line. Consistently, there was no association between the presence of anti-PLA2R antibodies and anti-podocyte reactivity (Supplemental Fig. 2H). IgM binding to podocytes was similar in a subset of patients with MN and healthy controls (median 23478 IQR 19498, 40098, vs median 23045 IQR 18003, 45861; p = 0.687).

Interestingly the MN samples that contained IgG reactive with GEnCs did not overlap with those samples that had IgG reactivity with the podocytes (Supplementary Fig. 5). These results suggest that MN patients have different patterns of autoreactivity against glomerular antigens, and that some patients may have autoantibodies reactive with endothelial antigens.

**Fig. 3:**
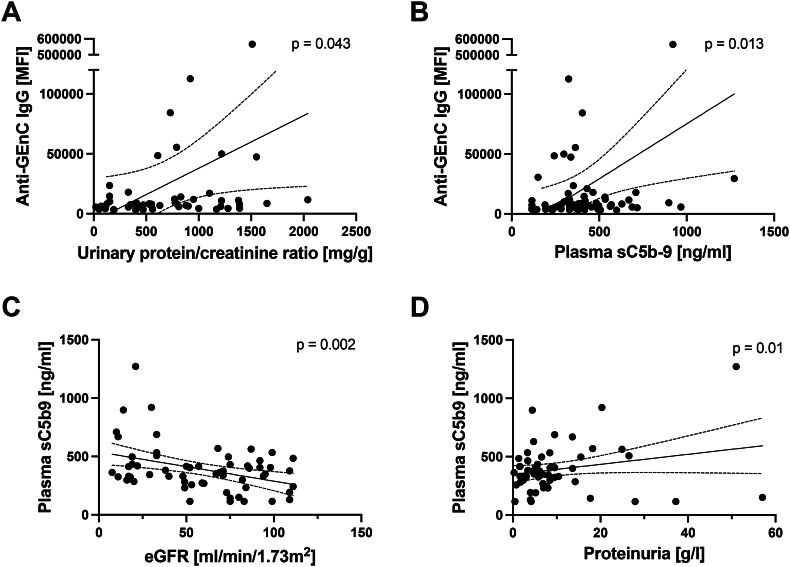
**Clinical correlations for the anti-endothelial antibodies and sC5b-9 levels.** A) The levels of anti-glomerular endothelial cell IgG (mean fluorescence intensity, or MFI) were significantly correlated with the urine protein/creatinine levels. B) The levels of anti-glomerular endothelial cell IgG were also significantly correlated with the plasma sC5b-9 levels. C) Plasma sC5b-9 levels were significantly correlated with the estimated glomerular filtration rate. D) Plasma sC5b-9 levels were significantly correlated with proteinuria levels.

We next correlated anti-GEnC and anti-podocyte reactivity with clinical data (Fig. 3 and Supplementary Fig. 6). Anti-GEnC IgG was significantly correlated with higher plasma sC5b-9 and higher proteinuria, a sign of disease severity. Plasma sC5b-9 was also significantly correlated with higher proteinuria and lower eGFR, both markers of disease severity.

### 3.4 Immunofluorescence staining of podocytes and GEnCs showed the same pattern as was seen by flow cytometry

**Fig. 4:**
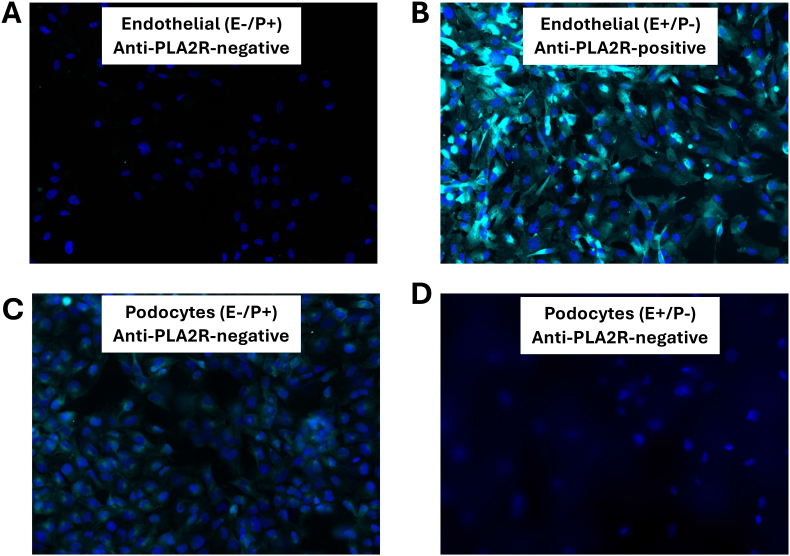
**Immunofluorescence microscopy of IgG binding to glomerular endothelial cells and podocytes.** Glomerular endothelial cells (A and B) and podocytes (C and D) were grown in culture, then they were incubated with serum samples from membranous nephropathy patients. Bound IgG was then examined by immunofluorescence microscopy. A) IgG was not detected on glomerular endothelial cells incubated with serum that had not shown positive binding by flow cytometry (E−). B) IgG was detected on the glomerular endothelial cells when they were incubated with samples that had also shown positive binding by flow cytometry (E+). C) IgG was detected on podocytes when they were incubated with serum samples that had shown positive binding by flow cytometry (P+). D) IgG was not seen when podocytes were incubated with serum that had not shown positive podocyte binding by flow cytometry (P-). The anti-PLA2R status for each sample is indicated in the legend.

Three patterns of IgG binding to GEnCs and podocytes were seen by flow cytometry: (1) GEnC binding but no podocyte binding (E+/P-), (2) podocyte binding and no GEnC binding (E−/P+), and (3) and negative reactivity with both cell types (E−/P-). To confirm these patterns, GEnCs were incubated with MN serum samples and examined using immunofluorescence microscopy (Fig. 4A–B). Strong signal for IgG on the GEnCs was seen when they were incubated with E+/P- serum, but no binding was seen when the cells were incubated with E−/P- or E−/P+ sera. No IgG bound to the cells when they were incubated with serum from healthy controls, or CKD patients (Data not shown). A similar experiment was performed using fixed podocytes (Fig. 4C–D). IgG bound to the cells when incubated with E−/P+ serum, but not when incubated with E+/P- or E−/P- serum. No IgG binding was seen when the cells were incubated with serum from healthy or CKD subjects (Data not shown). On the GEnCs, IgG staining was seen on the entire cell body. IgG reactive with the podocytes clustered on certain areas of the cell.

### 3.5 Western blot analysis demonstrates binding of IgG in MN samples to endothelial proteins

**Fig. 5:**
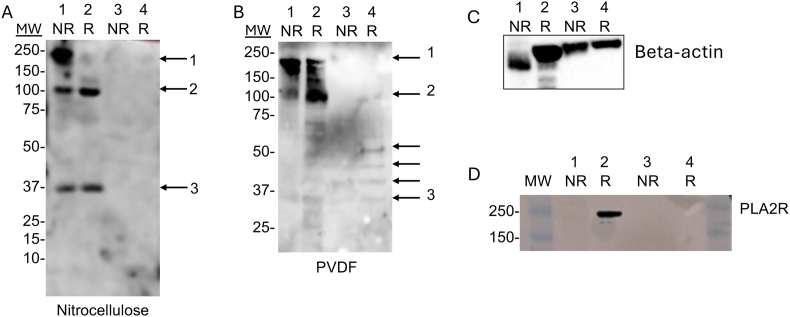
**Western blot analysis of human glomerular and endothelial cell lysates using serum from membranous nephropathy patients.** Glomerular extracts (1 and 2) and glomerular endothelial cells (3 and 4) were separated by gel electrophoresis and then transferred to A) nitrocellulose or B) polyvinylidene difluoride (PVDF). Samples were either non-reduced (NR) or they were reduced (R) by boiling with 2-mercaptoethanol. The blots were then probed with pooled serum from patients with membranous nephropathy. Bands can be seen in the glomerular extracts on both membranes (arrows), although they were better visualized on the PVDF membrane. Molecular weights (MW) are indicated to the left of each blot. The membranes were re-probed with an antibody to C) beta actin and D) PLA2R. C and D show the re-probed PVDF membrane.

We performed Western blot analysis in which protein lysates from whole glomerular extracts and GEnCs were probed with serum samples from MN patients who had shown anti-GEnC reactivity in the flow cytometry experiments. We examined non-reduced and reduced samples, and we also probed the samples after transfer to PVDF and nitrocellulose membranes (Fig. 5A and B). IgG in the MN serum clearly reacted with three bands in the glomerular extract. Several bands were also seen in the GEnC lysate, and they were best visualized in the reduced sample transferred to PVDF membrane. The blots were re-probed with antibodies to beta actin and PLA2R. PLA2R was seen in the whole glomerular extracts, but not in the GEnC lysate.

### 3.6 Systemic autoantibody titers against cardiolipin are similar in MN patients and controls

**Fig. 6:**
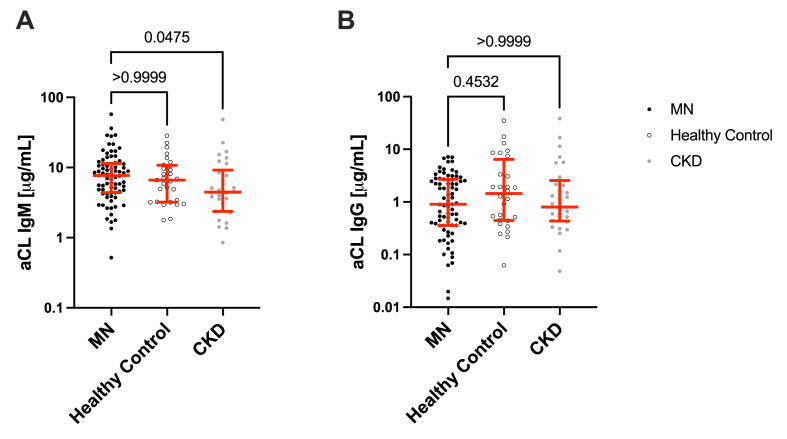
**Anti-cardiolipin antibodies IgM and IgG levels in membranous nephropathy patients.** Anti-cardiolipin IgM and IgG were measured by an enzyme-linked immunosorbent assay. The levels of anti-cardiolipin antibodies are shown on the Y axis. The horizontal red line shows the median, and whiskers denote the interquartile range.

Next, we tested whether immunoglobulins binding to GEnCs or podocytes could be natural antibodies. We previously found that levels of aCL IgM are higher in patients with FSGS than in healthy controls or in patients with non-proteinuric CKD [13]. We also found that aCL IgM binds to injured endothelial cells and can activate complement on the cells [13]. To examine whether the same is true in MN, we measured aCL IgG and IgM in serum samples from patients with active MN, and in healthy control subjects, as a marker of circulating natural antibodies. There was wide variability in aCL IgG and IgM reactivity (Fig. 6A–B). The titers of serum aCL IgG were not significantly different from healthy controls and CKD, but levels of IgM were higher in MN than in CKD. aCL antibodies did not significantly correlate with IgG binding to GEnCs or podocytes (Supplementary Fig. 6). No thrombotic events were clinically noted for any of the patients included in this study.

## 4 Discussion

MN is caused by autoantibodies reactive with podocyte antigens, and investigators have previously reported that complement activation fragments are elevated in both the plasma and urine of affected patients [12]. Although it is well known that C3 is deposited in the glomeruli of MN biopsies, detection of complement biomarkers in the plasma is notable, as fragments generated by activation on podocytes might be expected to pass into the urinary collecting system [10]. Furthermore, anti-PLA2R antibodies are usually IgG4. This subclass does not activate the classical pathway of complement, although anti-PLA2R antibodies may activate the lectin pathway [8,23]. In the current study, we found that complement activation fragments are elevated in the plasma of MN patients, including markers of classical/lectin (C4a) and terminal (C5b-9) complement pathway activation. Using cell-based assays, we found that distinct subgroups of MN patients had IgG that was reactive with GEnCs or with podocytes, and that anti-GEnC antibodies were associated with a worse MN phenotype. IgG in the serum of MN patients reacts with proteins expressed by the GEnCs, although we have not yet identified the target proteins.

We have previously reported that natural aCL antibodies react with epitopes displayed on injured endothelial cells [24]. These antibodies are associated with endovascular complement activation and might explain the presence of complement fragments in plasma, so we hypothesized that a similar phenomenon might occur in MN. We found that aCL IgG antibodies correlated with plasma C3a and with proteinuria, but that they did not correlate with anti-GEnC or anti-podocyte reactivity. Anti-GEnC antibodies could contribute to glomerular injury in MN by activation of the complement system on the capillary wall. Several recent studies have shown that C3a can mediated changes in podocyte morphology and cause proteinuria in MN [25,26]. The auto-antibodies could also cause injury by cross-linking target antigens on endothelial cells or by ligating Fc receptors on immune cells [27].

Immortalized cell lines often lose some of the phenotypic characteristics of their parent cells, but the segregation of the E+/P- and E−/P+ results supports the specificity of the cell-based assays. These findings also seem to be MN specific, as we did not see any reactivity with either cell type using healthy and disease control samples. Using samples that contained GEnC-reactive IgG by flow cytometry, we also detected IgG binding to the cells by immunofluorescence microscopy. Similarly, binding of IgG to podocytes by flow cytometry matched the results using immunofluorescence microscopy. There was an association between anti-GEnC reactivity and urine protein/creatinine levels. Higher levels of anti-GEnC IgG also associated with higher levels of plasma sC5b-9, linking the presence of these antibodies with complement activation fragments in plasma. No clinical correlations were seen for the anti-podocyte IgG detected by the cell-based assay. Anti-PLA2R status did not correlate with either anti-GEnC or anti-podocyte IgG.

Anti-PLA2R antibodies are seen in approximately 70 % of MN patients, and our results suggest that a subset of MN patients also produce IgG reactive with GEnC antigens. The podocyte cell line used in this study does not express PLA2R, so a small number of the patients also expressed IgG reactive with a non-PLA2R podocyte antigen. There was no association between the presence of anti-PLA2R antibodies and positivity in either of these cell-based assays. Based on these findings, MN encompasses a variety of autoimmune responses against targets on different glomerular cell types. The heterogeneous repertoire of autoantibodies may help to explain the variability in clinical outcomes seen among patients. We previously reported that patients with idiopathic nephrotic syndrome had higher levels of aCL antibodies, and the levels were inversely correlated with kidney function [13]. The level of aCL antibodies in the MN samples did not correlate with anti-GEnC reactivity, however, indicating that the flow cytometry assay is detecting autoantibodies that are reactive with different target antigens on the cells.

The results in this study align with a prior publication which reported that complement fragments are elevated in the plasma of MN patients [12]. It has long been known that sC5b-9 is elevated in the urine of patients with MN, and that this may be a marker of immunologically active disease [11,21,22]. Complement activation fragments in plasma may be indicators of anti-GEnC antibodies, and the level of sC5b-9 did correlate with the presence of anti-GEnC antibodies (Fig. 3B). It will be important to perform longitudinal studies in which the prognostic importance of these antibodies as well as plasma markers of complement activation are examined. As more patients with MN are treated with B cell targeted therapies, it will also be interesting to examine the effects of these treatments on autoreactive antibody levels and complement fragments in plasma. Surprisingly, factor H levels were higher in the MN patients, even though one might predict that the protein to be lost in the urine of proteinuric patients. Factor H is a complement regulator, and it is not clear how higher levels affect disease activity, but levels did not significantly correlate with markers of complement activation.

A limitation of these experiments is that the cell line which we used does not express PLA2R, so the cell-based assays probably do not capture the full spectrum of auto-reactivity in MN. It is challenging to create stable cell lines that fully recapitulate the phenotype of their parent cell types, a problem that is particularly acute in podocyte studies [28]. Importantly, we have not yet identified the target GEnC antigens, although several candidate proteins were seen by Western blot analysis. Future experiments can explore the identity of these proteins. Importantly, however, the target antigens could also be phospholipids or glycans, and future studies should also consider these possibilities [13]. Some kidney disease patients have natural antibodies that react with cardiolipin, but that does not appear to be the target of the auto-reactive IgG in the MN samples that we tested.

It will also be important to confirm our findings in additional patients, and to analyze samples from a sufficient number of patients to perform multivariate analysis and control for confounding factors, such as age, gender, and renal function. With additional samples, we can also examine the isotypes and complement-activating potential of the anti-GEnC antibodies. These efforts are ongoing. Finally, primary MN is considered a disease of autoimmunity against podocytes, and endothelial changes are generally not prominent. Indeed, subendothelial or mesangial immune deposits are suggestive of secondary disease. MN can lead to glomerulosclerosis, however, demonstrating that the disease can affect the entire glomerulus. Furthermore, recent studies in minimal change disease have shown that standard histologic analyses may not detect subtle endothelial changes or pathologically important IgG deposits [[29], [30], [31]].

In conclusion, we have found that patients with MN have elevated systemic and local complement activation and that a subset of patients have autoantibodies that are reactive with GEnCs *in vitro*. The presence of these antibodies appears to be specific for MN, and they may contribute to glomerular damage as they were correlated to clinical markers of disease severity. Future studies should explore whether these antibodies are of prognostic significance. In addition, identification of target antigens on endothelial cells may help investigators to test whether the antibodies are pathogenic.

## CRediT authorship contribution statement

**Vojtech Petr:** Writing – original draft, Investigation, Formal analysis. **Shrey Purohit:** Investigation, Formal analysis. **Felix Poppelaars:** Writing – review & editing, Investigation. **Brandon Renner:** Writing – review & editing, Methodology, Investigation. **Jennifer Laskowski:** Investigation. **Russell Whelan:** Investigation. **Liudmila Kulik:** Investigation. **Jessica Kendrick:** Methodology, Investigation. **Ashley Frazer-Abel:** Investigation. **Diana Jalal:** Writing – review & editing, Investigation. **Barbara Marcolin:** Investigation. **Isabelle Schmelzer:** Investigation. **Hanna Debiec:** Investigation. **Pierre Ronco:** Writing – review & editing, Investigation. **Moin A. Saleem:** Writing – review & editing, Investigation. **Simon C. Satchell:** Investigation. **Joshua M. Thurman:** Writing – original draft, Funding acquisition, Formal analysis, Conceptualization.

## Funding

This work was supported by 10.13039/100000002National Institutes of Health Grants R01DK076690 (JMT), DK133240 (JMT and DJ), DK138960 (JMT), and DK130255 (JMT and JK).

## Declaration of competing interest

The authors declare the following financial interests/personal relationships which may be considered as potential competing interests:Joshua M. Thurman reports financial support was provided by National Institutes of Health. Joshua M. Thurman reports a relationship with Q32 Bio Inc that includes: consulting or advisory and equity or stocks. Felix Poppelaars reports a relationship with Alnylam Pharmaceuticals Inc that includes: consulting or advisory. If there are other authors, they declare that they have no known competing financial interests or personal relationships that could have appeared to influence the work reported in this paper.

## Data Availability

Data will be made available on request.
