# Supplementary material for: Autoantibodies reactive with glomerular endothelial cells and podocytes in patients with membranous nephropathy
Source: J Transl Autoimmun. 2025 Dec 9;12:100342. doi: 10.1016/j.jtauto.2025.100342 (PMC12767797; doi:10.1016/j.jtauto.2025.100342)
Supplement: Multimedia component 1 [file mmc1.pdf]

Supplementary Figure 1

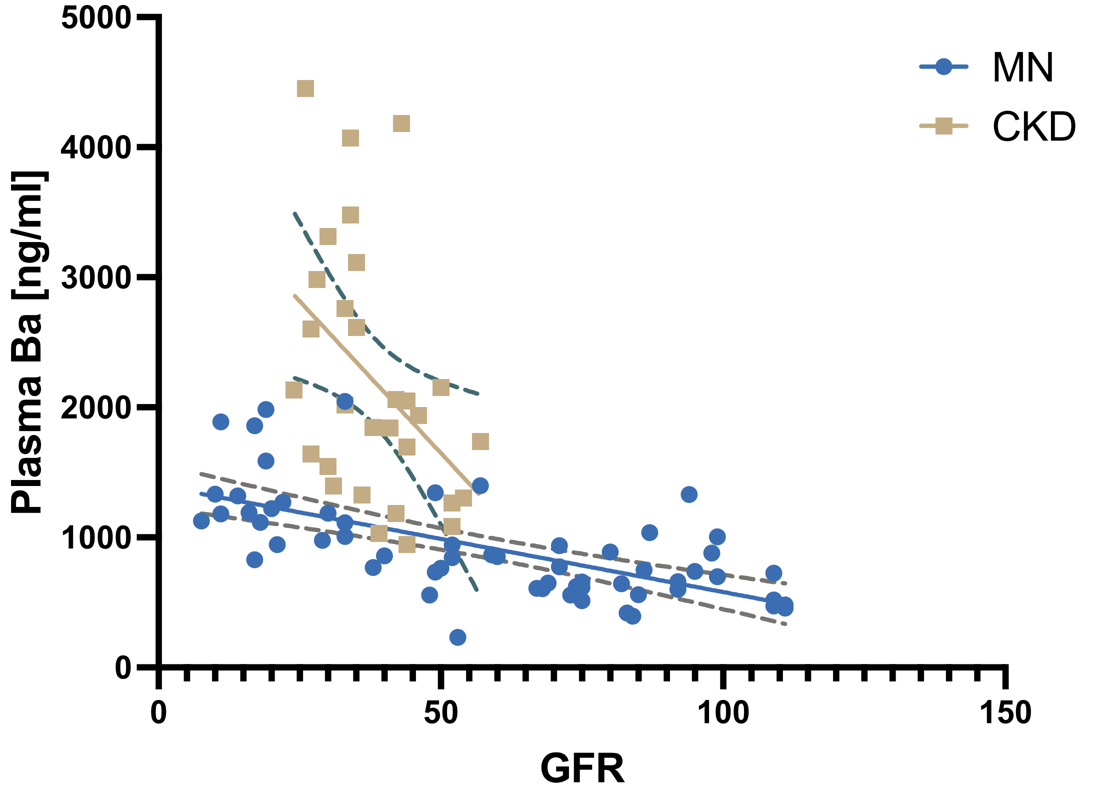

Supplementary Figure 1. Plasma Ba is lower in membranous nephropathy than in CKD despite similar eGFRs.

Supplementary Figure 2

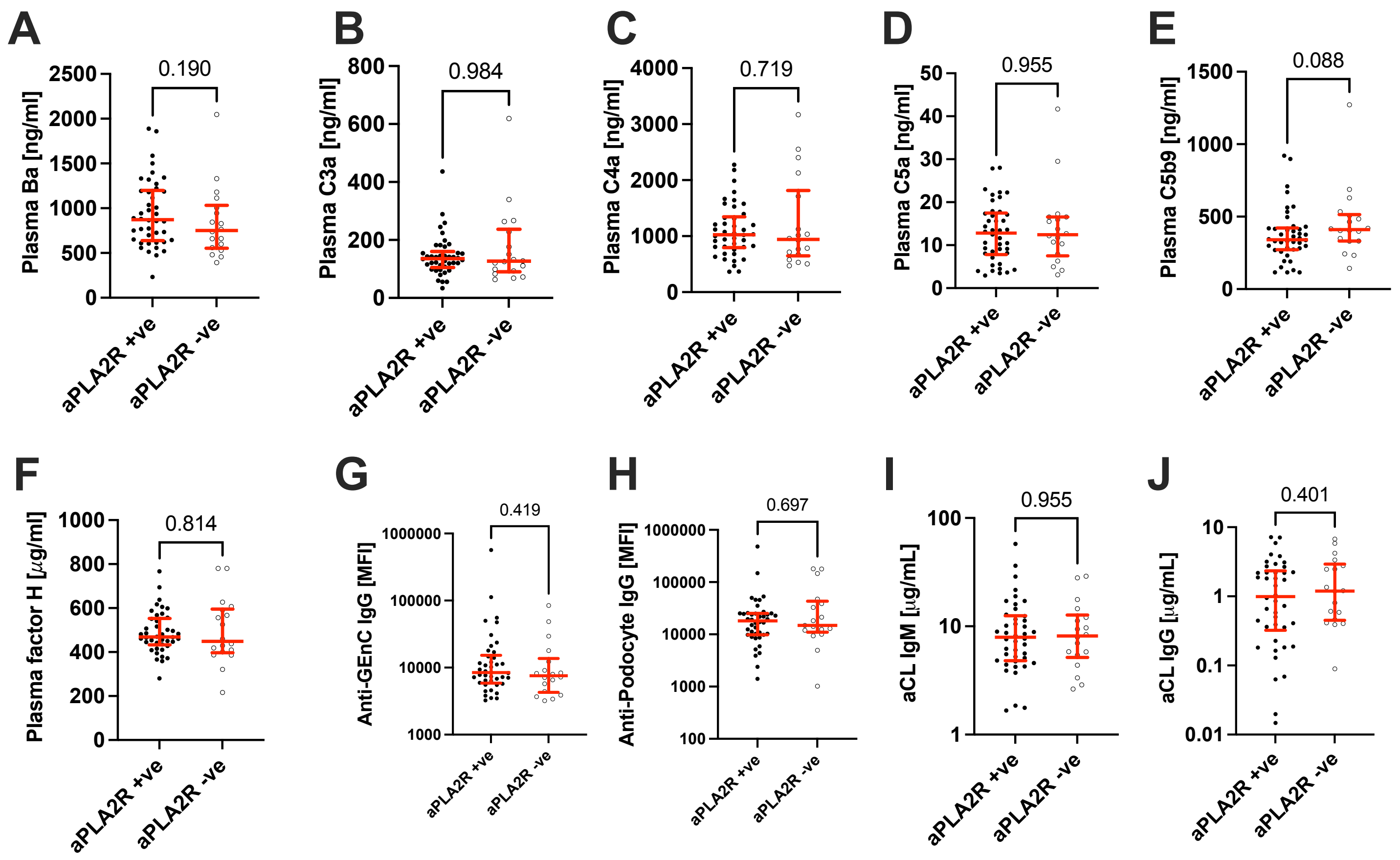

Supplementary Figure 2. Complement fragments and factors, anti-cardiolipin antibodies and IgG binding podocytes and endothelial cells according to anti-PLA2R antibody positivity/negativity. (A) Plasma fragment Ba, (B) plasma fragment C3a, (C) plasma fragment C4a, (D) plasma fragment C5a, (E) plasma C5b-9, (F) plasma factor H, (G) anti-endothelial IgG, (H) anti-podocyte IgG, (I) anti-cardiolipin IgM, and (J) anti-cardiolipin IgG.

Supplementary Figure 3

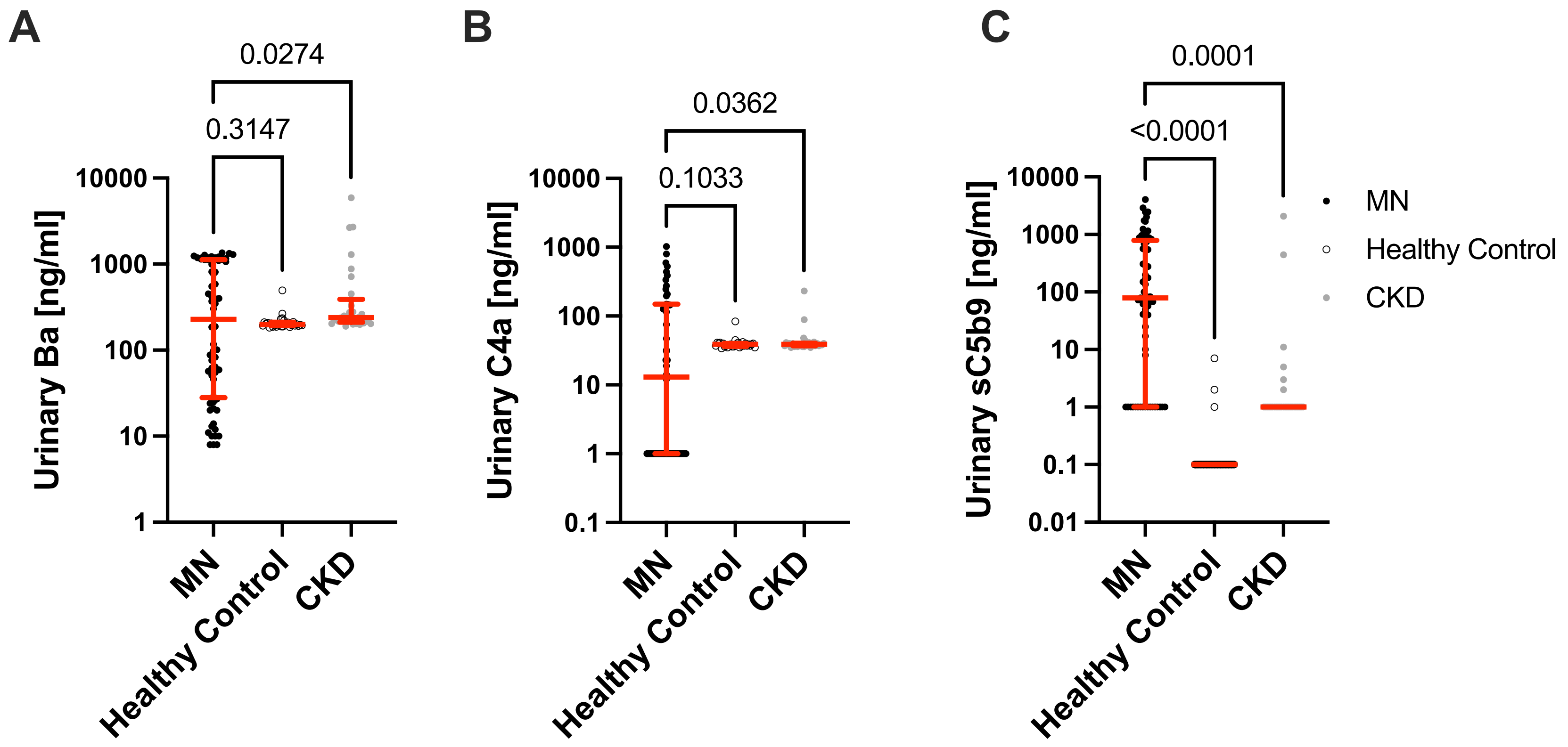

Supplementary Figure 3. Urinary complement measurements. (A) Urinary Ba is significantly higher in Ba subjects compared to chronic kidney disease (CKD), but similar to healthy controls, (B) Urinary C4a is significantly lower in membranous nephropathy (MN) subjects compared to CKD, (C) Urinary C5b-9 is significantly higher in MN compared to both healthy controls and CKD. Individual data points are showed in MN, healthy control, and CKD patient groups (X axis), Y axis shows respective analytes, horizontal red line denotes median, whiskers denote interquartile range, statistical testing was carried out by Kruskal-Wallis test, with post-hoc testing showing differences between MN and healthy controls, and MN and CKD.

Supplementary Figure 4

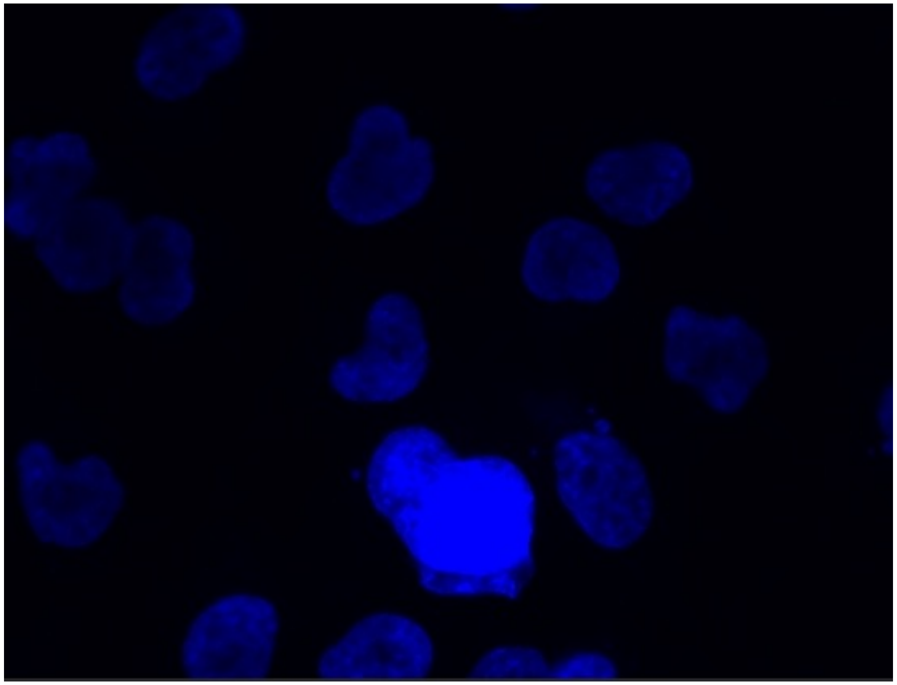

Supplementary Figure 4. Immunofluorescence showing that cultured podocytes do not express PLA2R.
The cultured podocyte cell lines does not express PLA2R. This is in contrast to podocytes in patients with membranous nephropathy.
DAPI – blue, IgG – green.

Supplementary Figure 5

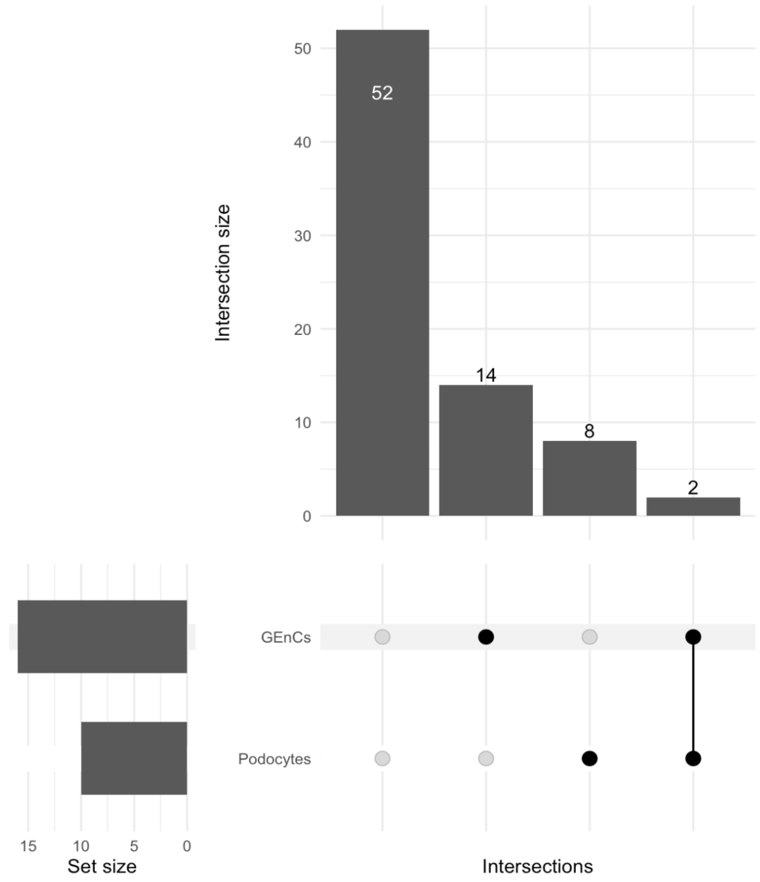

Supplementary Figure 5. Upset plot of positivity of binding to glomerular endothelial cells (GEnCs) and podocytes. Positivity was defined MFI above mean + 2*SD of MFI of healthy controls for GEnCs and podocytes. Only 2 patients sera exhibited positive binding to both cell lines.

Supplementary Figure 6

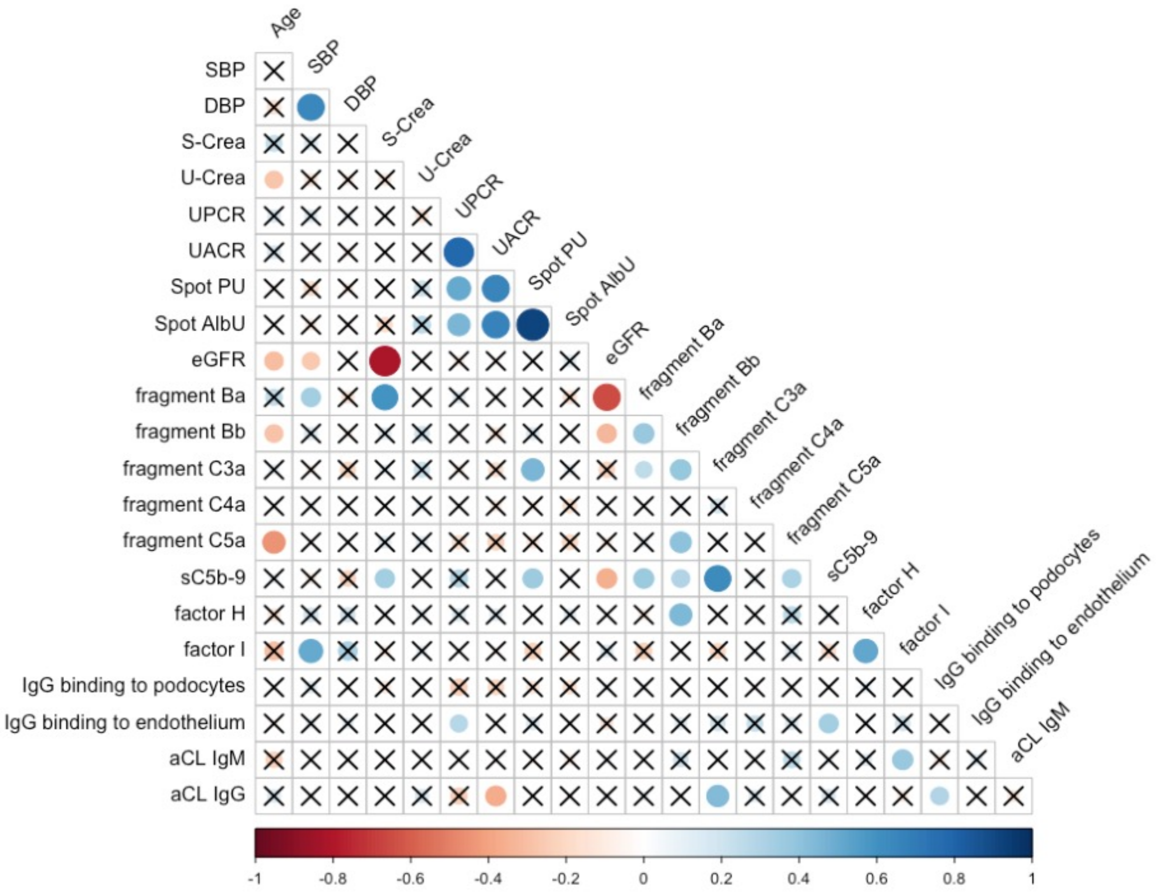

Supplementary Figure 6. Correlation matrix. Correlation matrix of tested continuous variables in the MN patients. Crossed squares denote statistically non-significant correlations, color of the circle corresponds to the direction and the strength of the correlation (red = negative correlation, blue = positive correlation, see the scale at the bottom of the figure). Size of the circle corresponds to statistical significance of the correlation.

Supplementary Table 1. Characteristics of healthy controls and CKD subjects.

Parameter	Healthy control (n = 30)	CKD G4/G5 (n = 30)
Age, [years] mean (SD)	38 (13)	59 (15)
Male sex, n (%)	6 (20)	18 (60)
eGFR [ml/min/1.73m^2], mean (SD)	82 (17)	37 (8)
Urinary albumin/creatinine ration [mg/g], mean (SD)	0.08 (0.09)	5 (14)
